# Matrix-Assisted Laser Desorption/Ionisation, Time-of-Flight Mass Spectrometry in Genomics Research

**DOI:** 10.1371/journal.pgen.0020100

**Published:** 2006-07-28

**Authors:** Jiannis Ragoussis, Gareth P Elvidge, Kulvinder Kaur, Stefano Colella

**Affiliations:** University College London, United Kingdom

## Abstract

The beginning of this millennium has seen dramatic advances in genomic research. Milestones such as the complete sequencing of the human genome and of many other species were achieved and complemented by the systematic discovery of variation at the single nucleotide (SNP) and whole segment (copy number polymorphism) level. Currently most genomics research efforts are concentrated on the production of whole genome functional annotations, as well as on mapping the epigenome by identifying the methylation status of CpGs, mainly in CpG islands, in different tissues. These recent advances have a major impact on the way genetic research is conducted and have accelerated the discovery of genetic factors contributing to disease. Technology was the critical driving force behind genomics projects: both the combination of Sanger sequencing with high-throughput capillary electrophoresis and the rapid advances in microarray technologies were keys to success. MALDI-TOF MS–based genome analysis represents a relative newcomer in this field. Can it establish itself as a long-term contributor to genetics research, or is it only suitable for niche areas and for laboratories with a passion for mass spectrometry? In this review, we will highlight the potential of MALDI-TOF MS–based tools for resequencing and for epigenetics research applications, as well as for classical complex genetic studies, allele quantification, and quantitative gene expression analysis. We will also identify the current limitations of this approach and attempt to place it in the context of other genome analysis technologies.

## Introduction

It is now commonly accepted that genetics plays a key role in the occurrence of common diseases, from diabetes and cancer to psychiatric disorders. At the same time, genetic approaches have been mostly successful in identifying the genetic component of Mendelian diseases. The construction of physical and genetic maps for the human genome [1–3] was followed by the complete human genome sequence and analysis [4]. This has allowed the experimental [5] and computational identification of transcribed sequences, and driven the execution of cost-effective positional cloning approaches, leading to the identification of about 1,000 genes causing Mendelian diseases. The completion of the human sequence was rapidly followed by the identification of the most common variations in the human genome, namely single nucleotide polymorphisms (SNPs), estimated at 10 million [6]. These major advances are being complemented by efforts aimed at the detection of the methylation status of CpG islands (Epigenome Project) [7] and by large-scale gene expression studies aiming to link gene expression patterns to genome variation [8].

The underlying force for these major advances is a collection of enabling technologies, spearheaded by Sanger sequencing combined with capillary electrophoresis and microarray-based technologies. Current efforts are concentrated towards dramatically improving the cost efficiency of whole genome sequencing approaches to achieve the goal of sequencing one genome a day at $100,000 each, and potentially for $10,000 or less [9].

One of the technologies that has been put forward as an alternative to the Sanger sequencing/capillary electrophoresis combination is matrix-assisted laser desorption/ionisation, time-of-flight mass spectrometry (MALDI-TOF MS). MALDI-TOF MS was introduced, and has established itself, as the tool of choice in proteomics applications, while the full potential for DNA analysis was demonstrated in 1995 [10] and for RNA in 1998 [11].

**Figure 1:**
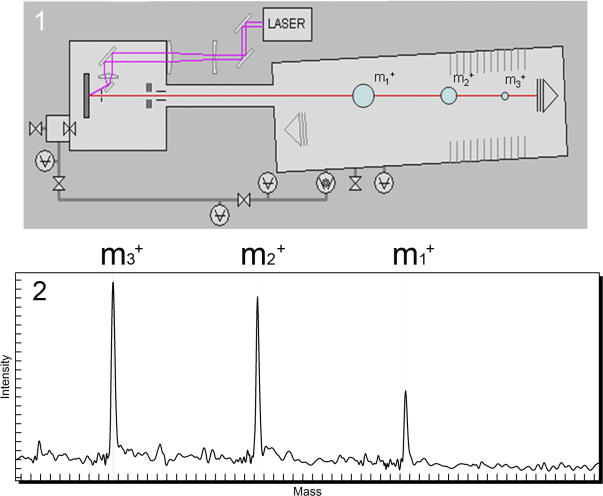
MALDI-TOF Mass Spectrometry The analyte is deposited on a matrix crystal containing spot. A laser is fired at the matrix leading to the desorption of the analytes and their ionisation. For the majority of DNA analyses, the matrix is designed to produce positive ions. The molecules accelerate into the flight tube where they fly towards the detector on the right. Low mass molecules arrive in a shorter time than heavier ones and molecules of different mass are thus separated (1). After data processing, a spectrum is produced with relative intensity on the *y*-axis and mass in Daltons on the *x*-axis (2).

In brief, for MALDI-TOF MS analysis single-stranded nucleic acid molecules of 3–29 bp in length (1,000–8,600 Dalton range) need to be generated and deposited on a matrix (e.g., 3-hydroxy picolinic acid). The analyte/matrix molecules are then irradiated by a laser inducing their desorption and ionisation, upon which the molecules pass through a flight tube connected to a detector on the other end (see Figure 1). Separation occurs by the time of flight, which is proportional to the mass of the individual molecules. The main advantage of the method is that it directly measures an intrinsic physical property of the molecules, namely their mass, and at a very high speed (about 100 μs). Limitations lie in the size of the DNA molecules that can be detected intact to less than 100 bp (due to size-dependent fragmentation during the MALDI process); and that the analytes must be free from ion adducts which lead to mass distortion. Efforts in the last ten years concentrated on developing simple, robust, homogeneous, and automatable assays suitable for a wide spectrum of genomic applications.

## SNP Genotyping

One of the most effective uses of MALDI-TOF MS in genome analysis is for SNP genotyping. Several methods have been developed for this application based on either a locus-specific PCR step followed by a primer extension, invader reaction, or hybridization steps with or without exonuclease digestion. The products are then detected by MALDI-TOF MS (reviewed in [12]). From all these possibilities, the most widely used are the GOOD assay [13], the PinPoint [14], the GenoSNIP (Bruker Daltonics, Billerica, Massachusetts, United States) and the MassEXTEND (Sequenom, San Diego, California, United States) [15]. The homogeneous MassEXTEND reaction (hME) represented an important step forward since it was the only truly homogeneous assay that is performed in one tube (well) using unmodified primers, and therefore best suited to high throughput analyses. The assay is based on generating PCR products, which are subsequently treated with shrimp alkaline phosphatase, followed by primer extension reactions using Thermosequenase (GE Healthcare, Bucks, United Kingdom) and stop mixes containing cocktails of ddNTPs and dNTPs. Adduct-forming ions are removed by adding an anion exchange resin. MALDI-TOF analysis is performed by piezo electrically dispensing 15 nl of the reaction products into silica-based chip arrays containing matrix spots (3-hydroxy picolinic acid) in a 384 format. A batch of ten of these chips can be loaded into a typical MALDI instrument (e.g., Bruker Biflex II/III or Autoflex I/II), allowing the analysis of 3,840 reactions in a few hours.

To be cost effective, the SNP assays must be multiplexed at a high level. Although 12-plex reactions have been demonstrated [16], designing multiplex PCR assays involves dealing with the competing parameters of specificity and uniform cycling conditions, and minimising interactions between primer sets.

**Figure 2:**
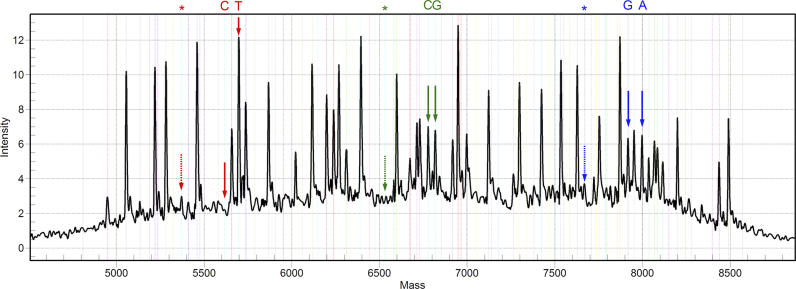
Example of a 29plex iPLEX Reaction For the iPLEX reactions, the primers are split into three groups according to the position of their respective mass peaks to use the whole spectrum. The concentrations of the three groups are adjusted so that peaks detected at the low mass end of the spectrum have a similar peak height to peaks at the high end of the spectrum. Three examples are highlighted in different colours in different areas of the spectrum. The unextended primer peak is marked by an asterisk and dotted arrow, while the other solid arrows indicate the two different alleles. In red, a homozygous genotype example is shown. In green and blue, two heterozygous alleles are shown. Note the similar peak heights at which extension products corresponding to the two alleles of an SNP are detected and their relative proximity. This proximity enables a higher accuracy to be achieved in the assay by applying the same level of local background subtraction and by eliminating misinterpretations resulting from small peaks, detected just above background, at the expected mass.

A step towards optimising multiplexed PCR design was achieved recently through the generation of a set of assay design algorithms that screen out primer interactions and perform simultaneous BLAT searches on the human genome for specificity optimisations, thus allowing automated design of primers for 20–30 plex reactions [17]. Sequenom optimised the primer extension reactions for MALDI-TOF MS by using acyclic mass modified base terminators that achieve at least 16 Dalton gaps between the four possible bases incorporated into a single base extension. Finally, noncomplementary bases are added to the 5′ of the extension primers to uniformly fill the 4,500–9,000 Dalton range of the spectrum. In parallel, the PCR and primer extension reaction conditions were optimised. As a result, this type of assay, called iPLEX, features a high design efficiency, low assay dropout rates, and high pass rates (about 95% in our hands) at typically the 26–29 plex level (Figure 2). At the same time, iPLEX is cost efficient, able to offer a very competitive cost in the “few cent” per genotype category, while the ability to process high sample numbers is retained. Due to the high efficiency PCR conditions, extra care is needed to avoid contaminations.

hME is suitable for large-scale collaborative projects requiring a mid-range number of SNPs (10–400) and high numbers of samples [18] and has also contributed to the first phase of the HapMap project. It is also suitable for projects requiring validation steps of ultra-high-throughput SNP-based disease association studies, as well as for genetic studies involving model organisms. A recent example is the development of SNP assays within the framework of the domestic dog genome sequencing project [19]. The ability to design and obtain primers within a very short time enabled us to perform rapid candidate gene analysis by hME using as few as two SNPs and 800 samples up to ten SNPs and 2,215 samples for association studies [20,21]. In addition, 200 SNPs have been analysed in 360 individuals to validate LD data [22]. The hME assay is the cost-effective choice where 2–9 plexes are sufficient on 384 samples or more, while iPLEX is very cost-effective when the assays involve multiples of 25–29 SNPs on 384 samples or more. In both cases, 7,680 samples can be processed within a few days.

Comparable alternative SNP genotyping technologies in terms of throughput include SNPStream from Beckman Coulter (Fullerton, California, United States) [23] and SNPlex from ABI (Foster City, California, United States) [24], both of which have the capability to multiplex 12–48 SNP assays in a 384-well format. SNPStream is based on primer extension and product capture on an array, while SNPlex is based on oligonucleotide ligation and PCR, followed by hybridization to fluorescent probes (ZipChute, Applied Biosystems) and separation by capillary electrophoresis. Equipment costs depend on the level of automation required, but are similar between Sequenom and ABI, while the Beckman Coulter equipment is less expensive. In terms of consumable cost/genotype, the iPLEX reaction is the most cost-efficient due to the combination of low SNP assay specific and general reagents costs. In terms of pass rate and accuracy, both MassEXTEND and SNPstream were used within the framework of the GenomEUtwin project [18], and comparable results were obtained. The MALDI-TOF–based methods have the advantage over other assays in that the user is in total control of the assay design process and is able to modify procedures and to develop his/her own protocols. The hME/iPLEX type assays have the additional advantage of requiring only unmodified primers, which allows for a minimal gap between SNP selection, design, and project start.

Determining SNP haplotypes is important for many research and clinical applications ranging from association studies to pharmacogenetics. Haplotypes are constructed either using pedigrees and statistical methods, or allele-specific PCR, or somatic cell hybrids [25]. Ivo Gut and colleagues combined allele-specific PCR with MALDI-TOF MS–based genotyping to determine haplotypes within a 2–4 kb range at high throughput [26]. Ding and Cantor took advantage of the sensitivity offered by the MALDI-TOF MS analysis and combined it with high efficiency PCR on highly diluted (3 pg) DNA samples to achieve molecular haplotyping [27]. The method, called M1-PCR, allows the direct determination of haplotypes within up to 24 kb of DNA. Although this latter method is able to generate haplotypes of longer distances than allele-specific PCR-based techniques, it is very sensitive to contamination.

Furthermore, MALDI-TOF MS–based genotyping has also found its way into many clinical genetic applications for SNP or known mutation detection such as in cystic fibrosis [28–31], and in other clinical applications involving the typing of bacteria and viruses [32,33].

## Quantitative DNA Analysis Applications

In MALDI-TOF MS analyses, the amount of analyte that is co-crystallized with the matrix and then ionized by laser excitation is proportional to the areas under the detected peaks. This allows the relative quantification of the molecules present in the analyte by calculating the peak area ratios. In this setting, variation in peak ratios in a large number of samples can be easily determined, and this quantitative data is one of the strengths of this method. The relative quantitative power of the approach has been used successfully to determine SNP allele frequencies in DNA pools [34–36] in allelotyping experiments. The area under the peaks corresponding to the two alleles is measured and used to determine a ratio, which is normalised to correct the skew due to different extension efficiencies using the ratio obtained from nonpooled genomic DNA (where we expect a 1:1 ratio between the two alleles). For accuracy, each measurement involves at least three technical replicates. MALDI-TOF MS emerged as one of the most accurate allelotyping techniques when compared with Pyrosequencing (Biotage, Uppsala, Sweden), TaqMan, and SNaPshot (Applied Biosystems) [37]. This method has been applied to disease association studies, particularly at a time when genotyping costs were relatively high [38,39]. Even if DNA pooling achieves considerable cost savings and has now been applied on high density Affymetrix GeneChip mapping arrays [40], generating good quality DNA pools requires careful adjustment of each individual DNA, particularly for large numbers of samples (thousands), and therefore becomes labour-intensive. This limitation may be overcome by introducing a whole genome amplification step, whereby the end DNA concentration of all samples after amplification is quite homogeneous [41]. The MALDI-TOF MS–based methods utilising DNA pools would be useful for allelotyping studies involving either candidate functional SNPs as shown in Butcher et al. [42] or to validate results from high-density SNP arrays [43].

## Detection of Indels Associated with MSI and Short Tandem Repeats

In addition to SNPs, another important polymorphism is represented by insertions and deletions (indels), some of which can be disease-causing mutations or associated with microsatellite repeat instability (MSI) in the form of length variation of short repetitive sequences. Repeat instability as a form of mutation is linked to a number of disease conditions [44] and cancer [45]. To date, indels and MSIs are detected using electrophoresis and DNA sequencing, which apart from being time-consuming and expensive, are not particularly sensitive in detecting quantitative differences. To solve this problem, MALDI-TOF MS has been applied in combination with assays utilising primer extension [46] or the generation of RNA fragments [47]. Bonk et al. [46] investigated MSIs linked to neoplastic lesions (colorectal tumours, cell lines, and normal control samples) and used primers designed to anneal directly at the 5′ of short mononucleotide repeats. Analysis of the primer extension products resulted in specific peak patterns, allowing the identification of unstable repeats in the tumour samples. Another approach was used by Sasayama et al. [47]. Briefly, in vitro transcription of PCR products was followed by cleavage at 5′ and 3′ of the indel site using molecular scissors and analysis of the resulting RNA fragment by MALDI-TOF, as demonstrated in the analysis of the *ApoE3* gene.

A related approach involving ribozymes was used by Krebs et al. [48] to type short tandem repeat sequences. The method was subsequently refined to utilize base specific cleavage of RNA molecules using RNase T1 [49] (see sequencing below).

## Gene Expression Analysis

Now that the HapMap project has produced working assays for about one million SNPs [50], future efforts will concentrate more on the identification and characterization of functional variants linked to phenotypes [51]. There is great interest in the identification of genetic elements influencing gene expression levels [52]: measuring relative expression levels of specific alleles carrying coding polymorphisms (with potential functional effects) and identifying the effect of DNA polymorphisms on the way hnRNA is spliced to produce mRNA. Once functional variants have been identified, it will be possible to link them to particular phenotypes, covering a broad spectrum from common disease to infection resistance and drug responses. The allelotyping approach described in the quantitative analysis applications can be adapted for allele-specific expression analysis involving coding SNPs or SNPs in UTRs. Briefly, the RNA is converted to cDNA by reverse transcription, followed by PCR of the segment containing the SNP and the interrogation of the SNP using a primer extension–based approach. In heterozygous individuals, we would expect two peaks in MALDI-TOF MS analysis. The area under the peaks corresponding to the two alleles is measured and used to determine a ratio similar to allelotyping [53]. The method has been used successfully to determine allele-specific expression at the *IL-8* locus [54]. In the absence of a transcribed SNP, it is possible to use the HaploChip technique [55] to assay for SNP alleles present near the transcription start site (within 1 kb) using Polymerase II loading as a marker for transcriptional activity. Antibodies against phosphorylated Pol II are used to immunoprecipitate cross-linked Pol II-DNA complexes. Upon reversal of the cross-link, the segment containing the transcription start site is amplified by PCR and the SNPs within the PCR product are interrogated by primer extension and MALDI-TOF MS. A distortion in the relative ratio of the two alleles indicates an imbalance in their respective levels of transcription.

**Figure 3:**
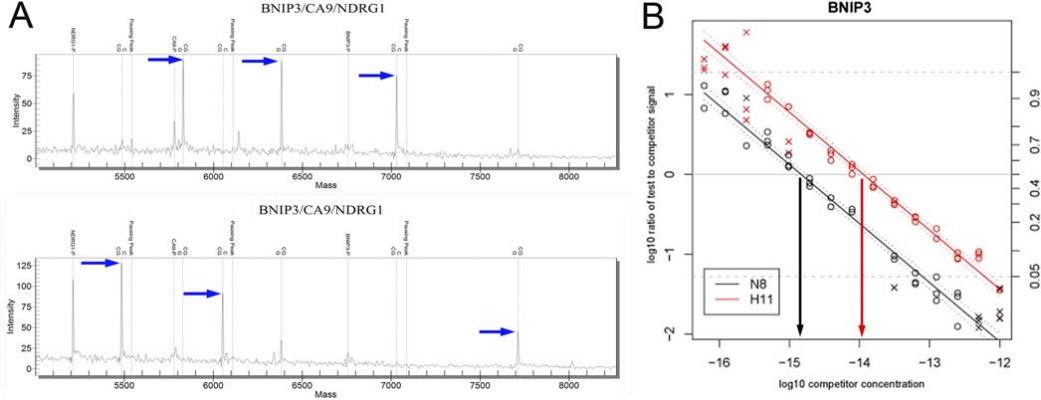
RC-PCR Experiment (A) A triplex assay for the genes *BNIP3, CA9,* and *NDRG1* is shown. Using a high competitor concentration (10^−12^M), competitor is predominantly PCR-amplified and only the primer extension products resulting from the competitor oligos (blue arrows, top graph) are detected. Using low competitor concentrations (10^−16^M), cDNA is predominantly PCR-amplified and only the primer extension products resulting from the cDNA are detected (blue arrows, bottom graph). The equivalence point, whereby an equal area of competitor and cDNA peaks is derived, would lie at a competitor concentration of between 10^−12^ M and 10^−16^ M. (B) Output of the TITAN software (http://www.well.ox.ac.uk/~tprice/titan) showing linear regression analysis of two samples (red and black) for BNIP3 expression. *x*-axis: competitor concentrations; *y*-axis (left) is the log 10 ratio of test (cDNA) to competitor signal. The equivalence point is given by the concentration at which *y* = 0, and is calculated by interpolation (black/red arrows). An approximated difference in expression is detected between the two samples.

Total levels of expression can be determined by the real competitive PCR technique (RC-PCR) [56]. This also involves converting RNA to cDNA and amplification of a segment by PCR (usually short amplicons 70–90 bp long). In addition, a competitor oligo of known concentration with the same sequence as the PCR region, apart from one single base close to the middle of the amplicon, is amplified in the same reaction. This type of sequence eliminates differences in the amplification rates between target and competitor and can be used to titrate the amount of target present in the total cDNA (see Figure 3). Typically 7–15 different concentrations of competitor are used and the results plotted in a titration curve. We have applied linear regression analysis using the software package TITAN, and this can be used to analyze data from any type of competitor PCR-based approach [57]. We have compared RC-PCR to real time PCR and found that the main advantage of RC-PCR was the relatively little optimisation required for each assay as compared with SYBR green–based real time PCR. Since the assay uses an internal standard, both competitor and cDNA are amplified under the same conditions and therefore the results are relatively insensitive to nonspecific PCR products or primer-dimers. In addition, the two rounds of amplification (PCR followed by primer extension) allow for two levels of specificity, ensuring that only specific products are detected by MALDI-TOF MS analysis. Disadvantages include the need to prepare dilution series; the addition of reaction components to each well can be complicated and laborious, ideally requiring the use of liquid handling systems and further post-PCR processing. The RC-PCR method is suited to the simultaneous analysis of 70–700 genes rather than one (or small number of) gene(s) in one (or few) sample(s) [57], particularly when combined with iPLEX. The method is also suitable for detecting copy number changes in the genome and is particularly suitable for the validation of expression-profiling results, or multiple signature genes [57]. Finally, primer extension–based techniques can be used effectively in multiplexed assays to identify RNA splice variants by interrogating diagnostic bases at the splice junctions [58].

## DNA and RNA Sequencing

The advantages of MALDI-TOF MS over gel-based separation methods can be summarised as high resolution, high speed, and an absence of compression zones. Considerable amount of work has gone into establishing this technology as a viable alternative to capillary electrophoresis of Sanger sequencing or enzymatic digestion reactions. Promising developments involving the use of solid phase capturable dideoxynucleotides, and the generation of RNA ladders from a DNA template have been reviewed extensively elsewhere [59,60]. Despite these developments, the read lengths obtained at a maximum of approximately 100 bp are not competitive when compared with Sanger sequencing/capillary electrophoresis [60,61]. As a result, alternative strategies are being pursued to increase throughput and cut costs. However, MALDI-TOF MS is well-suited to analysing RNA [62,63], which is more stable than DNA under MALDI conditions. In RNA analysis applications, this can be applied to identify post-transcriptional modifications in ribosomal RNA. This has been investigated in the context of resistance towards antibiotics mediated through post-transcriptional modification of rRNA using RNase digestion–based protocols [64]. Similar RNase digestion–based protocols have also been used to investigate RNA secondary structure to optimise the sequence of antisense oligonucleotides [65]. The assay involves using oligodeoxynucleotides that are used to form RNA–oligodeoxynucleotides duplexes which are subsequently recognised and digested by RNaseH. MALDI-TOF MS is the method of choice to analyse the resulting fragments.

**Figure 4:**
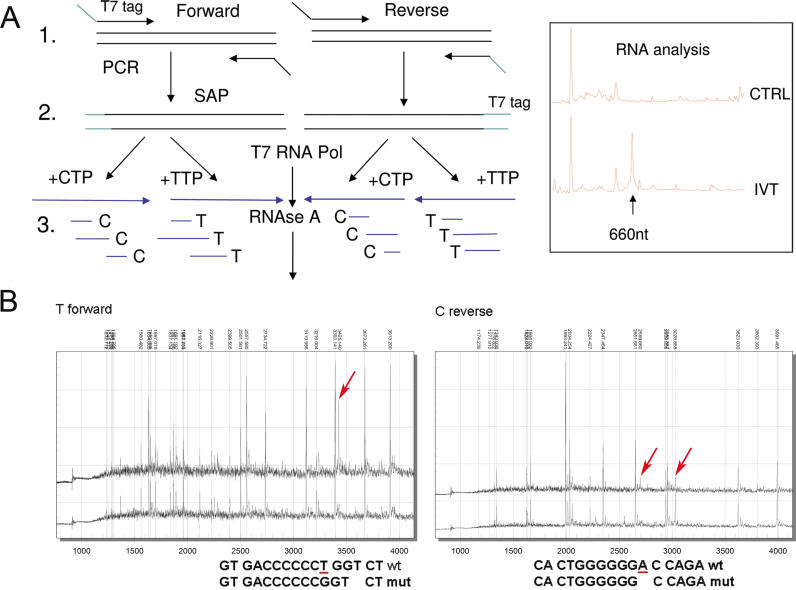
Base Specific Cleavage for Mutation Detection (A) Illustration of the MassCLEAVE procedure. (A1) PCR using one primer with a T7 RNA polymerase tag and one with a universal tag. (A2) In vitro transcription using T7 RNA polymerase and a mixture of dNTPs and NTPs. (A3) Cleavage by RNAse A. RNAse A cleaves at pyrimidines. By adding one of the pyrimidines as dCTP or dTTP only, the positions containing CTP or TTP are cleaved by RNAse A. Four cleavage reactions are set up in total. Box to the right of (A): Bioanalyser trace showing a full-length 660 bp RNA produced by IVT (IVT) compared with negative control (CTRL). The final steps involve sample conditioning and analysis by MALDI-TOF MS. Other possibilities include RNAseT1, which cleaves at a G. For methylation analysis, only the two reverse reactions are required. (B) Spectra and interpretation: A 396-bp fragment containing exon 6 of the *EXT1* gene was analysed by MassCLEAVE to identify a heterozygous deletion of a T at 160 bp. The informative T-forward and C-reverse reaction spectra are shown, whereby the spectrum produced by the sample under investigation is at the top and the control underneath. The numbers at the top indicate expected peak positions along the spectrum and are indicators of reaction quality. In the T-forward reaction, a new peak corresponding to the A1C6G3T1-composed primer appears at 3,425 Dalton (indicated by a red arrow). In the C-reverse reaction, a new peak at 2,699 Daltons is observed, corresponding to the T1G6C1 fragment, while the height of the 3,028 Dalton peak corresponding to the normal A1T1G6C1 fragment is reduced. The relevant normal and mutation-containing fragments are indicated and the deleted T (or A in reverse) is underlined in red. In total, 11/15 mutations in *EXT1* exon 6 and *EXT2* exon 5 (associated with hereditary multiple exostosis) were detectable with this method.

For DNA analysis, methods have been developed that involve in vitro transcription of DNA into RNA. RNA fragments are generated by base specific cleavage and analysed by MALDI-TOF MS, with the aim of identifying basepair changes in the sequence as compared with a computationally predicted outcome [66–69] (Figure 4A). These methods (for example MassCLEAVE (hMC) by Sequenom) are suited to resequencing projects for SNP and frameshift mutations detection (see Figure 4B) as well as SNP discovery using PCR products with a length of 200-1500 bp. More complex mutations involving longer indels or combinations thereof are difficult to determine correctly without further improvements in the algorithms (K. Kaur and J. Ragoussis, unpublished data). Although further bioinformatic optimisations have been incorporated which allow reactions to be multiplexed, the wide use of capillary electrophoresis to analyse Sanger-sequencing reaction products combined with the wealth of algorithms developed to interpret electropherograms and to detect mutations mean that it is likely that MALDI-TOF MS–based sequencing will remain a niche method. Considerable investment will also be required to compete against microarray-based resequencing, proven to be effective in detecting point mutations [70]. However, there is one exciting application that is a result of the efforts to develop MALDI-TOF MS–based sequencing approaches and that is methylation detection.

## DNA Methylation Analysis

DNA methylation at CpG sites in the genome contributes to several inherited human genetic diseases [71], and it has been postulated to contribute in a significant way to complex disease susceptibility [72]. The involvement of DNA methylation in gene expression regulation, genome stability, and imprinting has prompted the development and implementation of the Human Epigenome Project. This project will generate a genome-wide DNA methylation profile of the human genome with the analysis of different individuals and multiple tissues [7]. The project is under way and the profile of the human major histocompatibility complex, the target of the Human Epigenome Project pilot phase, has been recently completed [73]. The identification of the epigenetic factors contributing to disease etiology needs high-throughput technologies to detect DNA methylation. Many technologies have been developed [74], but only some of them lend themselves to high-throughput application. Among these, several are based on the detection of C/T SNPs that are chemically induced at CpG sites in the genome sequence by bisulfite treatment (where a C will be detected if methylation is present) [75]. Direct sequencing has been used, and several technologies developed for SNP detection have been adapted to bisulfite treatment–based methylation detection: including single nucleotide primer extension (SNuPE) combined with dot-blot [76] or with ion pair reverse phase high performance liquid chromatography (IP RP HPLC) [77], capillary electrophoresis [78,79], pyrosequencing technology [78,80,81], probe-based [82], and melting curve–based real-time PCR methods [83].

Whole genome approaches have been used to globally profile methylation levels including restriction landmark genomic scanning [84] and microarray-based approaches [85]. Methylation has been successfully detected as a chemically induced SNP after bisulfite treatment with specific oligo arrays [86] or using a methylated DNA positive (^me^C antibody) or negative (digestion with methylation-sensitive enzymes) selection approach on BAC arrays and/or tiling arrays for fine methylation mapping [87,88]. More recently, the Golden Gate genotyping assay on the BeadArray platform (Illumina, San Diego, California, United States) has been successfully adapted to detect methylation [89]. All microarray-based approaches allow the global mapping of overall methylation variation, but they do not offer high-resolution detection at the level of individual CpG sites. Furthermore, these approaches work on the assumption of extended methylation in the region interrogated, which could limit the detection sensitivity when complex unknown patterns of DNA methylation are present.

**Figure 5:**
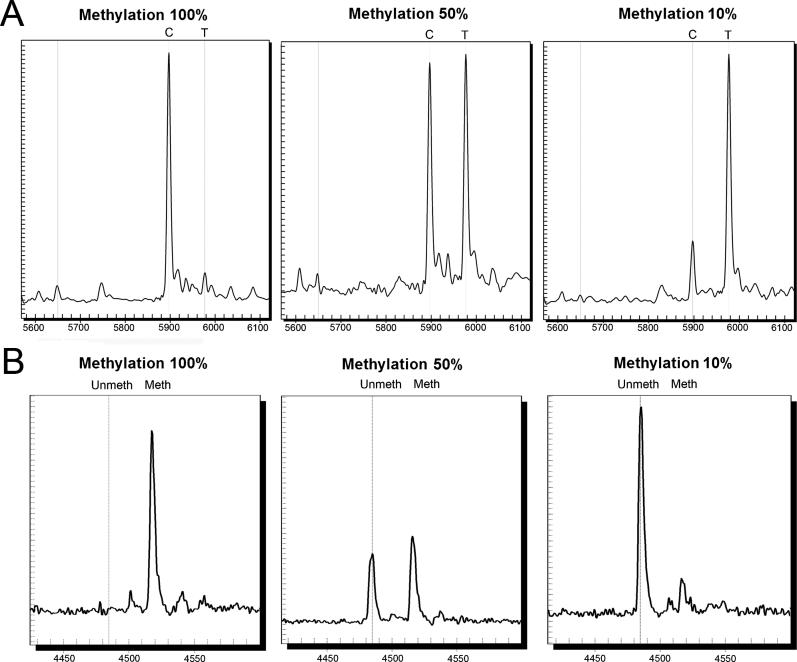
Methylation Detection Using MALDI-TOF MS MALDI-TOF MS spectra for the detection of different DNA methylation levels (examples shown: 100% methylated DNA; 50:50 mix of methylated and unmethylated DNA; 10% methylated DNA samples mixed with 90% unmethylated DNA) after bisulfite treatment in specific CpG positions in the promoter of *INK4A* (p16) gene (specific amplicon mapping to Chromosome 9: 21964960–21965128 on the Human Genome May 2004 assembly). (A) Primer extension approach using iPLEX chemistry to detect DNA methylation at CpG 2 (Chromosome 9: 21965105–21965106), C call corresponding to the methylated and T call to the nonmethylated alleles. (B) MassCLEAVE assay using T-reverse specific cleavage, detection of the methylation levels at CpG 10–11 (Chromosome 9: 21964989–21964992 on the Human Genome May 2004 assembly). Fragments detected on the reverse strand are as follows: 4484.85 Dalton 5OH-AAAAAAACCACAAT-3p for the unmethylated (Unmeth) and 4516.84 Dalton 5OH-AAAAAAACCGCGAT-3p with both CpG sites methylated (Meth).

MALDI-TOF MS approaches, originally developed for SNP genotyping and SNP discovery, have been adapted to the detection of the chemically induced SNP present in the DNA after bisulfite treatment. A primer extension–based approach in the form of the GOOD assay has been successfully developed and used for the quantification of methylation levels [90]. Based on the same principle, the protocol for allelotyping analysis as implemented in the MassARRAY system (Sequenom) (see “Quantitative DNA analysis applications”) can be applied to primer extension reactions. We have used both the MassEXTEND protocol (hME) and the iPLEX protocol, to successfully detect and quantify methylation at specific CpG positions (Figure 5A; S. Colella and J. Ragoussis, unpublished data). The major disadvantage of primer extension–based approaches, irrespective of the detection platform, is the assay design limitation within CpG rich regions, as it is better to avoid having a CpG site (and therefore a potential SNP) in the sequence complementary to the extension primer. This problem can be circumvented now by using peptide nucleic acid probe hybridization, instead of primer extension, to interrogate methylated CpGs. MALDI-TOF analysis of successfully hybridized peptide nucleic acids was shown to produce quantitative data at high sensitivity [91].

An alternative approach is based on sequencing by base specific cleavage as described in the previous section. This method generates different cleavage patterns for the methylated and unmethylated CpG positions present in the fragment analysed. This approach for methylation detection was developed by Schatz et al*.* using a guanosine-specific cleavage by RNase T1 [92]. More recently, Ehrich et al*.* have developed an assay using RNase A [69], based on modification of the hMC SNP discovery assay. In this method only the reverse strand is used as a template for the in vitro transcription, followed by the two base specific fragmentations (T-reaction examples in Figure 5B). The spectra obtained from the two reactions are combined and allow the quantification of methylation at CpG sites present in the amplicon analyzed. The final CpG coverage achieved depends on the amplicon size (a maximum of ~800 bp) and the specific sequence content. The average number of CpG sites detected in a single amplicon is comparable to sequencing-based approaches.

The RNA cleavage–based approach, e.g., hMC, is quite unique and competitive in rapidly generating a comprehensive CpG island quantitative methylation map. The method is comparable to direct sequencing in terms of throughput, but superior in terms of quantification. The quantification in turn is comparable to a combined cloning and sequencing approach, with the advantage that RNA cleavage is much easier and faster to perform. The RNA cleavage offers the possibility of detecting more CpG sites in a single amplicon, when compared with pyrosequencing, and, furthermore, it is independent from the extension primer design issues. One possible limitation is that some CpG sites cannot be analysed if they are in fragments that have a molecular weight outside the mass detection range. Array-based methods offer the highest throughput in detecting multiple promoter regions in a single experiment, but at present they do not compare to the detailed profile that can be generated with the RNA cleavage–based MALDI-TOF MS approaches. In conclusion, the MALDI-TOF MS resequencing–based method for methylation detection offers a level of resolution that enables high-throughput quantitative analysis of full CpG islands. Data generated with this approach can lead to a better definition of complex methylation patterns of specific regions and provide a better understanding of the effect of specific methylation events in the human genome**.**

## Discussion

It is clear that MALDI-TOF MS is a versatile tool for genomic research, allowing the analysis of DNA as well as RNA fragments. Due to the high sensitivity and ability to quantify, it is also suitable for clinical diagnostic use. In terms of number of applications, it is comparable to a DNA sequencer. The main advantages are that the technology, by measuring the mass of DNA molecules, allows a high degree of freedom for the individual investigator to conceive assays where the products can be analysed by MALDI-TOF MS. Even as a complete commercially available genomic applications system, as offered by Sequenom or Bruker Daltonics, it is relatively straightforward to apply custom assay design. The other advantage is the high sample number that can be analysed in each run, up to 3,840 samples at a time. This makes applications such as allele-specific gene expression and high-resolution methylation analysis very attractive for the study of functional polymorphisms and epigenetic modifications. Since all applications so far are based on a PCR step, developing new tools to assess PCR primers (and where applicable extension primers) interactions could lead to further improvements in efficient assay design and increase the level of multiplexing, particularly in genotyping assays.

For genotyping applications, the level of multiplexing that can be achieved with current methodology has limits. For example, the currently used part of the spectrum is in the 4,500–9,000 Dalton range. If we leave the PCR multiplexing issues aside, it is theoretically possible to accommodate 93 peak trios of an iPLEX reaction (extension primer and two alleles) at 16 Dalton intervals, thus increasing the number of reactions that can be multiplexed at current resolution levels. This level of multiplexing would result in significant genotyping cost reductions.

An additional problem being addressed is that the instruments (mass spectrometers such as Bruker Biflex II/III or Autoflex and spotting devices) themselves are expensive and maintenance-intensive, while high throughput applications require the use of liquid handling systems. Both Bruker and Sequenom offer compact, benchtop instruments in the form of the Microflex (Bruker Daltonics) and the Compact (Sequenom) to address the cost (and maintenance) issue.

One important advantage of MALDI-TOF MS that has not been fully utilized is the sensitivity of the technology. For example, no more than 15 nl of analyte is used on a MassARRAY chip. This low level of reaction volume would achieve high cost efficiency and could be realized with miniaturized nanoliter scale devices (for an example see [93], for a review see [94]). For example, the iPLEX assay takes place in 5 μl or 10 μl volume/sample, where the costs, in broad terms, are equally divided among PCR, extension reaction, and chip. It is easy to calculate that such an approach will result in significant savings by cutting two-thirds of the cost by a factor of ten or more. All methods described so far, such as resequencing and expression profiling, are amenable to the same cost reduction principle, and this would allow compact MALDI-TOF MS instruments to be integrated with miniaturized liquid handling systems.

So which applications are going to be most widely used? We think that the genotyping assays (such as iPLEX) are going to be the main applications. This is because current whole genome association studies using hundreds of thousands of SNPs will produce genomic segments or genes with disease associations requiring further rounds of replication in additional sample collections. Furthermore, additional SNPs must be investigated in order to identify the ones directly associated with disease. These downstream studies will require genotyping technologies that offer high-cost efficiency at medium levels of multiplexing and high sample throughput, currently a role fulfilled by the MALDI-TOF MS–based techniques. If further instrument development produces compact, simple-to-use, and lower-maintenance equipment (for example the benchtop MALDI microMX from Waters, [Milford, Massachusetts, United States]), then the added benefits of being able to perform a wide spectrum of additional assays will be a clear advantage. The ability to use the same platform to perform genomic studies beyond SNP genotyping, such as methylation detection, quantitative gene expression, and allele-specific expression, will be a bonus when equipment decisions have to be made for genomics and functional genomics laboratories.

Furthermore, development of compact systems such as the Microflex or MALDI microMX that can be adapted for use in genomic or proteomic applications, will allow MALDI-TOF MS to expand its position in the future as a versatile platform for basic research and clinical diagnostic applications alike. 

## Supporting Information

### Accession Numbers

The accession numbers for the following genes from the EntrezGene databank (http://srs.pku.edu.cn/srs/srs) are: *ApoE3* (348), *BNIP3* (664), *CA9* (768), *EXT1* (2131), *IL-8* (3576), *INK4A* (1029), and *NDRG1* (10397).

## Abbreviations

hMC: homogeneous MassCLEAVE
hME: homogeneous MassEXTEND reaction (or protocol)
indels: insertions and deletions
MALDI-TOF MS: matrix-assisted laser desorption/ionisation time-of-flight mass spectrometry
MSI: microsatellite instability
RC-PCR: real competitive PCR
SNP: single nucleotide polymorphism
